# Supplementary material for: Machine Learning to Understand the Immune-Inflammatory Pathways in Fibromyalgia
Source: Int J Mol Sci. 2019 Aug 29;20(17):4231. doi: 10.3390/ijms20174231 (PMC6747258; doi:10.3390/ijms20174231)
Supplement: Supplementary file 1 [file ijms-20-04231-s001.pdf]

Int. J. Mol. Sci. **2019**, *20*, 4231,doi:10.3390/ijms20174231

Electronic supplementary files

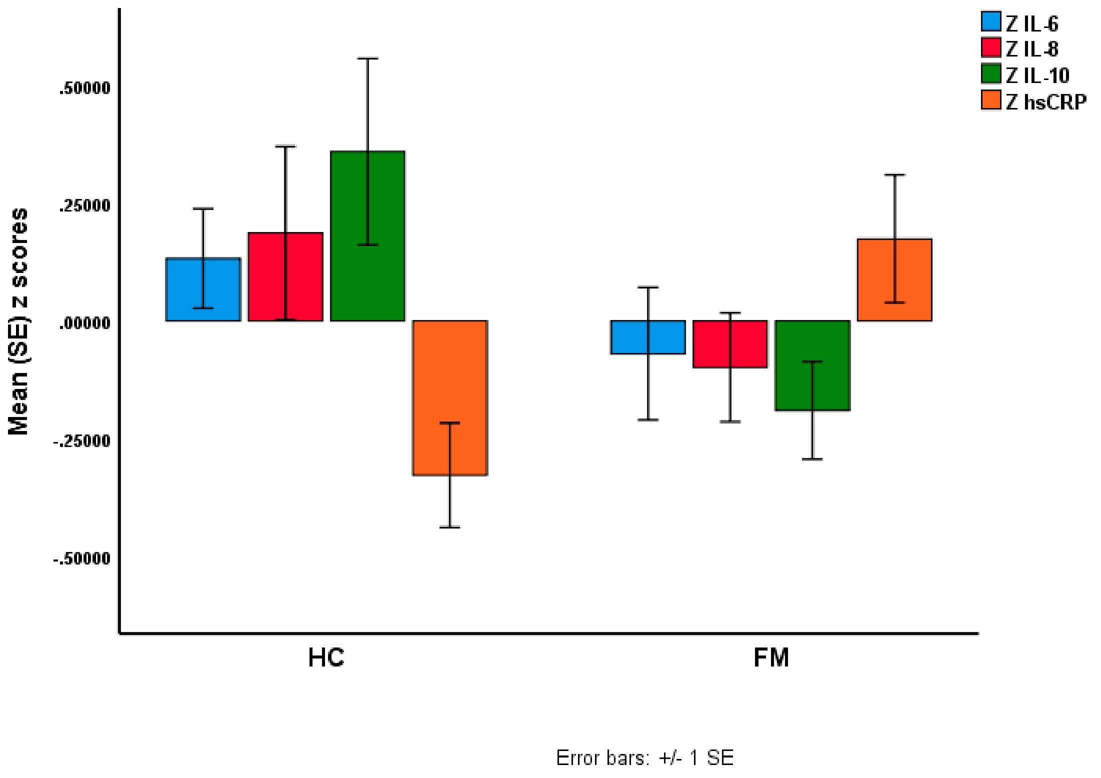

ESF1 Figure S1. Bars graph of the z transformed values of the assessed biomarkers per each group.

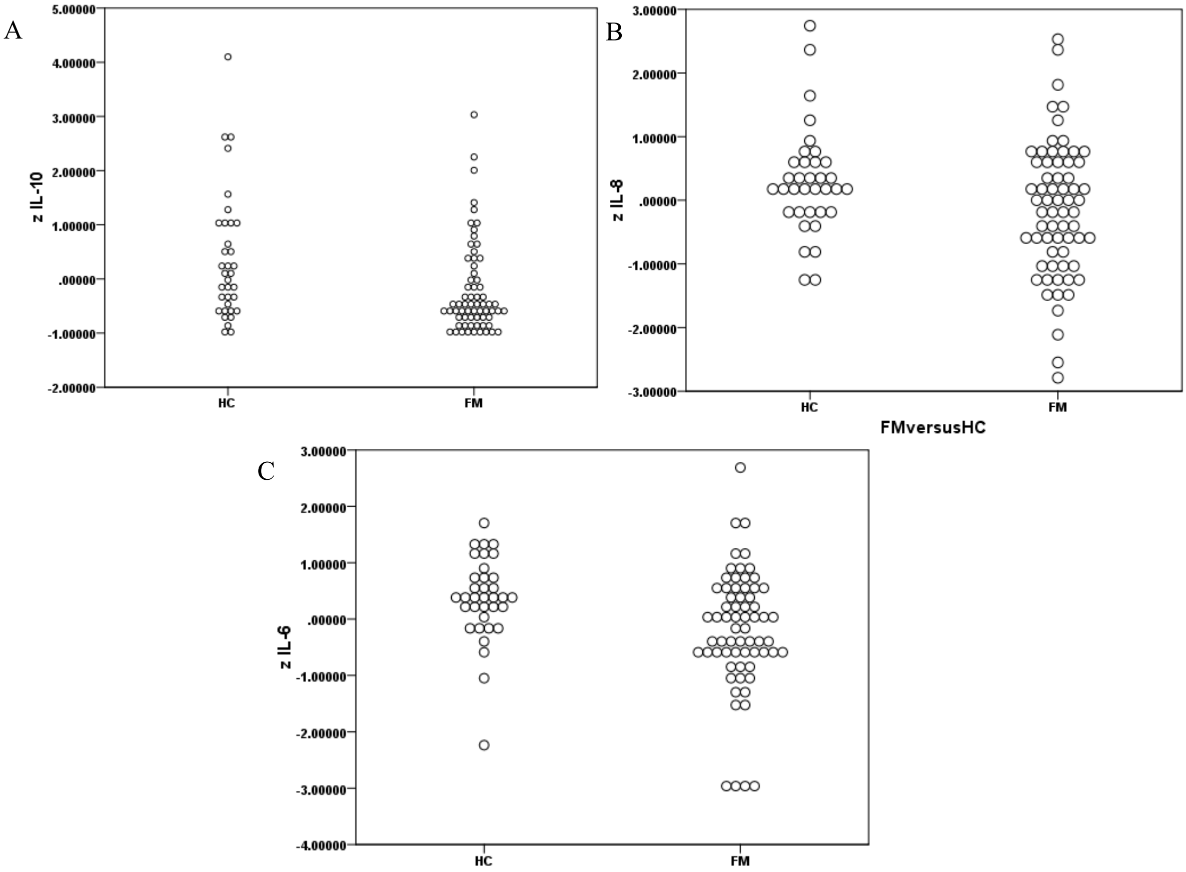

ESF1 Figure S2. Plots graph of z transformed IL-10 (**A**), CXCL-8 (**B**) and IL-6 (**C**) levels of healthy controls (HC) and fibromyalgia patients (FM).
